# The relationship between sex life satisfaction and job stress of married nurses

**DOI:** 10.1186/1756-0500-5-445

**Published:** 2012-08-19

**Authors:** Hsiu-Hui Lee, For-Wey Lung, Pei-Rong Lee, Wei-Tsung Kao, Yu-Lan Lee

**Affiliations:** 1 Nursing Department, Kaohsiung Armed Forces General Hospital, Kaohsiung, Taiwan; 2 National Kaohsiung Marine University, Kaohsiung, Taiwan; 3 Songde Branch, Taipei City Hospital, No.309, Songde Rd., Xinyi Dist., Taipei City 11080, Taiwan; 4 Department of Psychiatry, National Defense Medical Center, Taipei, Taiwan; 5 Nursing Department, Tzu-Chi University, Hualien, Taiwan; 6 Psychiatric Department, Kaohsiung Armed Forces General Hospital, Kaohsiung, Taiwan

**Keywords:** Effort-reward imbalance, Mental health, Sexual desires, Sex life satisfaction

## Abstract

**Background:**

The purpose of this study was to investigate the relationship among work stress, sex life satisfaction, and mental health of married nurses. Demographic information, work stress, sex life satisfaction, sexual desire and mental health measured using the Chinese Health Questionnaire, data were collected from 100 married nurses in Taiwan.

**Findings:**

Sex life satisfaction and age were negatively correlated, but sex life satisfaction and sexual desire were positively correlated. The mental health of over-committed nursing staff was not affected. Higher reward for effort was positively correlated with sex life satisfaction.

**Conclusions:**

No matter whether job stress was high or low, receiving a higher reward for effort led to better sex life satisfaction, which had a satisfying positive effect on the nurses' lives. To improve nursing care quality at the hospital, nursing administrators should assist nurses in confronting work stress via positive adjustment, which is associated with the nurses’ sexual harmony, and quality of life.

## Findings

In the globalized environment of the twenty-first century, the demand for higher quality medical services grows steadily while health technology and policies evolve continuously. These trends challenge service efficacy and the quality of the healthcare system, as well as health professionals [1], and consequently, the effort-reward imbalance has became a more and more serious and prevalent problem among nursing professionals in both developed and developing countries [2]. Many studies have shown that nursing staff, compared to other healthcare staff, experience a high level of stress [3-8]. Studies of the impact of nursing shifts on female sexual functioning have found that sexual dysfunction, age and BMI are risk factors that can lead to sexual dysfunction and have a negative impact on the perceived health status of nursing staff [5]. Therefore, having a satisfying sex life is crucial for maintaining a good quality of life [9].

The Effort-Reward Imbalance (ERI) model proposed by Siegrist [10] is a recent social exchange theory which states that a disturbed balance between effort and reward, i.e., great effort but little reward, can induce negative health impacts, and elicits negative emotions [11]. The feeling of disappointment and not being appreciated is associated with strain reactions in the autonomic nervous system [11]. Under such conditions, high ERI have been found to be predictive of onset of severe depressive symptoms at one-year [12] and five-years follow-up [13].

Sexual satisfaction can be generally defined as “the degree to which an individual is satisfied or happy with the sexual aspect of his or her relationship. Lin [5], in Taiwan, studied the impact of nursing shift work on female sexual functioning and discovered that 51.9% of nursing staff doing shift work experienced one sexual desire disorder, 10.9% experienced a sexual satisfaction disorder, and 68.85% experienced more than one sexual function disorder. Stress is unavoidable in a modern work environment, but few studies have examined the association between work stress and sexual satisfaction of nursing staff in Taiwan.

Therefore, the two main purposes of this study were to investigate 1) the association among work stress, sexual satisfaction and sexual desire of nursing staff, and 2) the effects of work stress on the mental health of nursing staff.

### Methods

The research protocol was approved by the institutional review board of Kaohsiung Armed Forces General Hospital, and is in accordance with the Declaration of Helskinki. Nursing staff from a teaching hospital in southern Taiwan were purposively sampled. To participate in this study, the respondents had to be a basic-level nursing staff (deputy head nurse or above were excluded), over 20 years old, a married female, and with more than three months of experience working in this hospital. Data were collected between September and October of 2009. Of the 106 questionnaires collected, 100 were valid (a 94.3% return rate). Informed consent was obtained from all participants after detailed explanation of the purpose of the study.

Participants had to provide data on demographic information, and completed Effort-Reward Imbalance Work Stress Scale (ERI-WSS) [10], Derogatis Sexual Satisfaction Scale [14], developed by Spector, Carey, & Steinberg [15] and translated into Chinese by Lin, then further modified by Lee, Chu, Ruan, Tzeng and Lung [16] and finally the Chinese Health Questionnaire (CHQ) [17].

Analysis of variance (ANOVA) was used to examine the effect of the four effort-reward imbalance groups on demographics, sexual desire, sexual satisfaction, and mental health. Linear regression analysis was further used to find the association between the respondents’ age, educational level, number of years of work experience, work stress, sexual satisfaction, and sexual desire. In addition, structural equation modeling was adopted to analyze the causal relationship among the variables.

### Results

The average age of the participants was 35.76 ± 6.43 years. All respondents had college or above education (100%). The distribution of the other demographic features is shown in Table 1.

**Table 1:** Demographic distribution (N = 100)

	**Mean ± SD or n (%)**
Age, years	35.76 ± 6.42
26-30	21(21%)
31-40	55(55%)
41-56	24(24%)
Number of children	
None	17(17%)
1	29(29%)
2	44(44%)
>2	10(10%)
Years of working experience	
0.5-1	1(1%)
>1-5	23(23%)
6-10	35(35%)
11-20	33(33%)
>20	8(8%)
Level of education (years)	14-18 (100%)
Having a religious belief	88(88%)
Department	
Psychiatry	17(17%)
Intensive care unit	16(16%)
Internal Medicine	15(15%)
Outpatient	13(13%)
Hemodialysis Center	10(10%)
General Medicine	10(10%)
Surgical	7(7%)
Operation Room	6(6%)
Gynecology	3(3%)
Emergency Room	3(3%)

Using the definition of Siegrist et al. [11], the upper tertile scores of effort and over-commitment were defined as high-risk conditions, and the effort–reward ratio greater than one was defined as a high-risk condition for imbalance. Therefore, respondents whose total score for intrinsic effort was in the upper one-third of the study population were classified as over-committed individuals. The Effort/Reward (E/R) ratio was defined as (the total effort score/the total reward score) x adjustment factor (6/11). If a respondent’s E/R value was greater than one, then the respondent was classified as having an effort-reward imbalance, i.e., experiencing higher work stress. The E/R value and the total intrinsic score data of the study respondents were calculated according to the above description, and the distribution of the questionnaires measured is shown in Table 2.

**Table 2:** Results of assessment variables with Effort - Reward Imbalance model, Sexual satisfaction, Sexual desires, CHQ

**Variables**	**Evaluation References**	**n**	**Mean ± SD or %**
Over-commitment			16.0 ± 2.6
	Reference value ≧17(High)	49	49%
	Reference value<17(General)	51	51%
Effort			18.1 ± 5.4
Reward			37.8 ± 3.6
Effort/Reward	E/R value>1 (Imbalanced group)	31	31%
	E/Rvalue ≦1	69	69%
	Low effort / High reward (EFF-REW+)	59	59%
	High effort / High reward (EFF + REW+)	30	30%
	Low effort / Low reward (EFF-REW-)	7	7%
	High effort / Low reward (EFF + REW-)	4	4%
Sexual satisfaction			26.2 ± 7.7
Sexual desires			33.2 ± 15.8
CHQ			2.7 ± 3.0
	CHQ <3	61	61%
	CHQ ≧ 3	39	39%

One-way ANOVA was used to investigate the association among effort-reward imbalance, over-commitment, sexual desire, sexual satisfaction and mental health, as shown in Table 3. Results showed significant differences between effort/reward conditions, sexual satisfaction and CHQ score (F = 9.42, p <0.001; F = 3.89, p = 0.011; F = 2.68, p = 0.050).

**Table 3:** One-way ANOVA test for associations between effort-reward imbalance and nursing staff’s over-commitment, sexual desire, sexual satisfaction and mental health

**Work stress type**	**Low effort/ High reward (Mean ± SD)**	**Low effort/ Low reward (Mean ± SD)**	**High effort/Low reward (Mean ± SD)**	**High effort/ Low reward (Mean ± SD)**	**F**	***p***
Over-commitment	15.22(2.04)	15.00(3.61)	19.75(2.87)	17.30(2.40)	9.42	<0.001
Sexual desire	32.76(14.46)	41.29(18.53)	25(11.75)	33.17(18.06)	0.98	0.405
Sexual satisfaction	26.31(6.94)	27.29(7.70)	37.50(9.11)	24.17(8.10)	3.89	0.011
CHQ	2.07(2.57)	2.43(2.51)	3.75(3.30)	3.83(3.45)	2.68	0.050

The effect of the nursing staff’s demographics, work stress, sexual desire and mental health on sexual satisfaction was using linear regression, as shown in Table 4. Parsimonious results showed that the older the married clinical nursing staff, the higher the sexual satisfaction (β = 0.699, p = 0.003), and the higher the sexual desire, the lower the sexual satisfaction (β = −0.312, p = 0.001).

**Table 4:** Parsimonious multiple linear regression analysis of nursing work stress and sexual satisfaction

**Variable**	**β**	**SE**	**p**
Age	0.70	0.26	0.003
Sexual desire	- 0.31	0.46	0.001

Dependent variable: sexual satisfaction.

**Figure 1:**
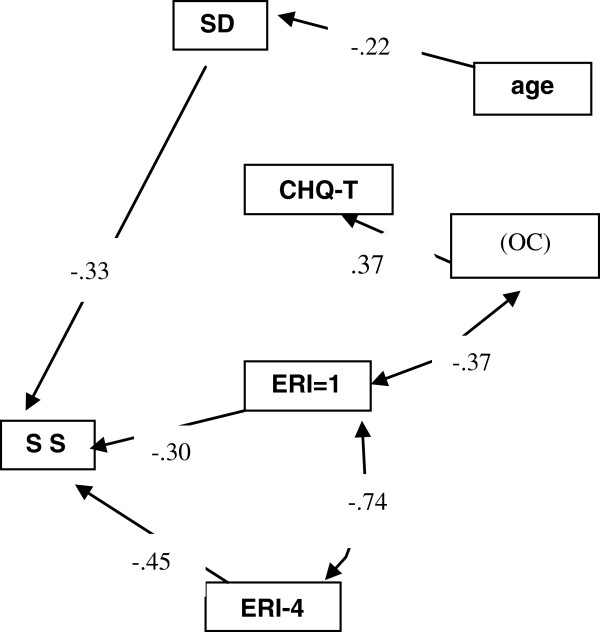
**The final robust parsimonious structural equation model of maternal bonding, age, sexual desire (SD), sexual satisfaction (SS), over-commitment (OC) and mental health (CHQ), ERI-1: Effort/ Reward (EFF-/REW+), ERI-4: Effort/Reward (EFF + REW+).** χ^2^ = 7.640; df = 14; p = 0.907; AGFI = 0.957; RMSEA <0.001.

The pathway relationship among the variables of interest was analyzed using structural equation modeling. The parsimonious model showed age, sexual desire, sex life satisfaction (SLS), over-commitment (OC) of work stress, high extrinsic effort/high reward (EFF + REW+), low extrinsic effort/high reward (EFF-REW+), and mental health (CHQ) had significant pathways. The model resulted in a p value of 0.907 (greater than 00.05), adjusted goodness-of-fit index (AGFI) of 0.957 (greater than 0.9) and root mean square error of approximation (RMSEA) of <0.001 (less than 0.08), showing a good fit, as seen in Figure 1.

The model showed age and sexual desire were negatively correlated (β = −0.22, p = 0.023). Sexual desire and high sexual satisfaction were positively correlated (the higher the sex life satisfaction score, the less satisfaction) (β = −0.33, P <0.001). Low effort/high reward and high effort/high reward with regard to work stress conditions were both positively correlated with sex life satisfaction (β = −0.32, P = 0.027; β = −0.42, P = 0.004). This finding showed a significant difference in sex life satisfaction with high reward, whether for high effort or low effort. Low effort/high reward and over-commitment were negatively correlated (β = −0.37, P <0.001). Nursing staff who were over-commitment were more likely to have worse mental health conditions (β = 0.37, P <0.001).

### Discussion

This study investigated the association between sexuality and work stress in married nursing staff using questionnaires. The average score of the nursing staff on the 11 questions on sex life satisfaction was 26.2, suggesting above average satisfaction. ANOVA and path coefficient results both suggested that low effort/high reward and high effort/high reward with regard to work pressure were positively correlated with sex life satisfaction. In other words, significant differences in sex life satisfaction were found with high reward, whether it was high effort or low effort. Since in the current society, both parents work in most families, thus the salary of the female nursing staff is also contributes to the family income, thus the satisfaction of the nursing staff also comes from the amount of the salary received. This finding also demonstrated that family income, among various living conditions, can affect sex life satisfaction [18], and satisfying the material aspect of life can optimize one's physical health [19]. In addition, greater sexual desire points toward more sexual satisfaction, and consequently an affectionate couple and a happy family. Various studies have also noted that the relationship and interaction of a couple can influence the level of sexual satisfaction. Furthermore, physical and emotional closeness are positively correlated with the occurrence of sexual activities [20,21], and thus a higher level of sexual satisfaction. Helstrom, Sorbom and Backstrom [22] proposed that two important factors influencing the female's satisfaction with her sex life are the absence of both serious life pressure and financial worry, which is consistent with our finding that sex life satisfaction is positively correlated with high reward.

Thirty-one percent of the respondents in this study were classified as having a work stress imbalance according to the ERI scale, compared to the prevalence of work stress among female laborers in Taiwan is 13.5% [23]. Therefore, we can say that nursing personnel experience greater work stress than other career women in Taiwan.

Furthermore, the nursing population in this study showed a higher degree of effort-reward imbalance than the populations of other studies. In this study, 49% of the respondents were classified as over-committed, which is higher than the 37% over-commitment rate of nursing staff from a general hospital [7]. Additionally, the mean score of 16 for over-commitment is also higher than the average for nurses found in European countries (mean 11.9 ~ 15.1) [24]. According to Siegrist, over-commitment is a pathological behavior mode. This type of person cannot mentally withdraw from work, and therefore stress responses are more likely to occur in this type of person. McGillis Hall and Kiesners [25] did a qualitative research on the work environment of nurses in Canada, and found that nurses reported problems of heavy workload and understaffing, causing them to feel helpless. Furthermore, these nurses are unable to provide the quality of care they wish due to the heavy workload, which effected their esteem [25], and over-committed individuals tends to have a strong desire for esteem [26]. When people put a great amount of mental effort in their jobs but do not receive the expected reward (such as respect or esteem), emotional exhaustion could arise [27], resulting in poor mental health.

A limitation of our study was that we did not measure actual sexual behaviors, instead, only self-perceived sexual desire and satisfaction of these nurses was measured. However, previous studies have found both in Europe and China that sexual satisfaction was associated with penile-vaginal intercourse for both women and men [28,29]. Additionally, although the results of this study comply with previous studies, the participants were recruited from the same hospital, thus the generalization of the results maybe limited.

Taken together, a high level of work stress among nursing staff can indirectly and negatively influence their mental health. Continuously imbalanced conditions would cause mental stress, stress responses, or illness. In a low reward situation, an individual would invest more work energy in exchange for better work control, but this measure could significantly damage one’s physical, mental health, and sexual satisfaction. Furthermore, although our linear regression results showed a positive association between age and sexual satisfaction, our structural equation model showed a negative association between sex and sexual desire, meaning those elder in age decrease in their level of sexual desire. Additionally, a negative association between sexual desire and sexual satisfaction was also found, showing those with greater sexual desire were less satisfied with their sexual lives. Thus the positive relationship between age and sexual satisfaction was mediated by the factor of sexual desire. If structural equation modeling was not used, this relationship would not have been found, thus shows the importance of structural equation modeling. Findings from this study can be used as a reference for nursing administrative policy-making and for hospitals (organizations) to ensure the nurses are not understaffed and are receiving the adequate payment for their effort. In order to provide high-quality patient care, work stress among the nursing staff has to be well handled.

## Competing interests

All authors have no conflict of interest to declare.

## Authors’ contributions

HHL and FWL conceived and supervised the study. HHL and PRL supervised the data collection process. WTK completed the analysis. YLL assisted with the data analysis and interpretation of the data. All authors contributed to the writing of the paper and approved of the final manuscript.

## References

[B1] Rong JR Chung UL The challenge facing nursing education: to develop clinical performance examination strategies in nursing practice J Nurs 2006 53 17 20 16475068

[B2] Hasselhorn HM Peter T Richard P Effort-reward imbalance among nurses in stable countries and in countries in transition Int J Occup Environ Health 2004 10 401 408 15702754 10.1179/oeh.2004.10.4.401

[B3] Cross W Moore A Ockerby S Clinical supervision of general nurses in a busy medical ward of a teaching hospital Contemp Nurse 2010 35 245 253 10.5172/conu.2010.35.2.245 20950204

[B4] Gelsema TI Doef M Maes S Akerboom S Verhoven C Job stress in the nursing profession: The influenec of organiztional and environmental conditions and job characteristics Int J Stress Manag 2005 12 222 239

[B5] Lin MY Effects of rotating-shift work on female sexual function in nurse- two hospitals study. Master Thesis 2008 Taipei, Taiwan: Graduate Institute of Public Health of Taipei Medical University

[B6] Peng M Liu Y Zhang A A study on literature in job stress of cancer ward nursing staff Nursing Magazine 2003 50 71 75

[B7] Tseng C A study on the relationship between work-related stress and physiological and mental health of nursing staff in medical centers. Master thesis 2004 Taipei, Taiwan: Health Care Organization Administration, College of Public Health, National Taiwan University

[B8] Tseng GH A study on the relationships among job strain, self evaluated health status and turnover tendency in hospital nursing staff in central Taiwan. Master Thesis 2005 Taichung, Taiwan: Graduate Institute of Healthcare Management of China Medical University

[B9] Brody S The relative health benefits of different sexual activities J Sex Med 2010 7 1336 1361 10.1111/j.1743-6109.2009.01677.x 20088868

[B10] Siegrist J Adverse health effect of high effort/low reward conditions J Occup Health Psychol 1996 1 27 41 9547031 10.1037//1076-8998.1.1.27

[B11] Siegrist J Starke D Chandola T Godin I Marmot M Niedhammer I Peter R The measurement of effort-reward imbalance at work: European comparisons Soc Sci Med 2004 58 1483 1499 10.1016/S0277-9536(03)00351-4 14759692

[B12] Wang JL Patten SB Currie S Sareen J Schmitz N A population-based longitudinal study on work environmental factors and the risk of major depressive disorder Am J Epidemiol 2012 epub ahead of print 10.1093/aje/kwr473 PMC3385158 22556191

[B13] Rugulies R Aust B Madsen IE Burr H Siegrist J Bültmann U Adverse psychosocial working conditions and risk of severe depressive symptoms. Do effects differ by occupational grade? Eur J Public Health 2012 epub ahead of print 10.1093/eurpub/cks071 22683769

[B14] Young M Denny G Luquis R Young T Correlates of sexual satisfaction in marriage Can J Hum Sex 1998 72 115 127

[B15] Spector IP Carey MP Steinberg L The sexual desire inventory: development, factor structure, and evidence of reliability J Sex Marital Ther 1996 22 175 190 10.1080/00926239608414655 8880651

[B16] Lee HH Chu YH Ruan FF Tzeng DS Lung FW Confirmatory factor analysis of the sexual desire inventory in patients with schizophrenia Taiwanese Journal of Psychiatry 2007 21 176 183

[B17] Cheng TA Wu JT Chong MY Williams P Internal consistency and factor structure of the Chinese Health Questionnaire Acta Psychiatr Scand 1990 82 304 308 10.1111/j.1600-0447.1990.tb01389.x 2260484

[B18] Huang HW Study on the relationship between sexual satisfaction and psycho-social adjustment on women after hysterectomy. Master Thesis 2004 Kaohsiung, Taiwan: Shu-Te University Academic Institutional Repository

[B19] Tseng Y A survey on job stress and mental health effects in nursing staff. Master's thesis 2004 Tainan, Taiwan: Institute for Environmental Studies, National Cheng Kung University

[B20] Carpenter LM Nathanson CA Kim YJ Physical women, emotional men: gender and Sexual satisfaction in midlife Arch Sex Behav 2009 38 87 107 10.1007/s10508-007-9215-y 17851747

[B21] Wu Z Chen H Huang X Lee T A study on sexual vitality and physiological diseases of the elderly people over 65 years-old Journal of Chinese Home Care 1998 8 72 79

[B22] Helstrom L Sorbom D Backstrom T Influence of partner relationship on sexuality after subtotal hysterectomy Acta Obstet Gynecol Scand 1995 74 142 146 10.3109/00016349509008924 7900511

[B23] Dai J Yang R Yeh W A survey on employees' perception of workplace health and safety in the area of Taiwan 2002 Taipei, Taiwan: Institute of Occupational Safety and Health

[B24] Hasselhorn HM Tackenberg P Peter R Next-Study Group Effort-reward imbalance among nurses in stable countries and in countries in transition Int J Occup Environ Health 2004 10 401 408 15702754 10.1179/oeh.2004.10.4.401

[B25] McGillis Hall L Kiesners D A narrative approach to understanding the nursing work environment in Canada Soc Sci Med 2005 61 2482 2491 10.1016/j.socscimed.2005.05.002 15964672

[B26] Tsutsumi A Kayaba K Nagami M Miki A Kawano Y Ohya Y Odagiri Y Shimomitsu T The effort-reward imbalance model: experience in Japanese working population J Occup Health 2002 44 398 407 10.1539/joh.44.398

[B27] Buunk BP Schaufeli WB Schaufeli WB, Maslach C, Marek T Professional burnout: a perspective from social comparison theory Professional Burnout: Recent Developments in Theory and Research 1993 Washington: Taylor & Francis 53 69

[B28] Tao P Brody S Sexual behavior predictors of satisfaction in a Chinese sample J Sex Med 2011 8 455 460 10.1111/j.1743-6109.2010.02129.x 21114768

[B29] Brody S Costa RM Satisfaction (sexual, life, relationship, and mental health) is associated directly with penile-vaginal intercourse but inversely with other sexual behavior frequencies J Sex Med 2009 6 1947 1954 10.1111/j.1743-6109.2009.01303.x 19453891

